# Psychological Assessment of Parenting Stress and Emotional Support: A Moderated Mediation Model of Psychological Distress and Well-Being Among Childbearing-Age Women with Thyroid Cancer

**DOI:** 10.3390/bs16091491

**Published:** 2026-08-26

**Authors:** Yinfeng Li, Min Peng, Yin-Ping Zhang

**Affiliations:** Faculty of Nursing, Xi’an Jiaotong University Health Science Center, Xi’an 710061, China; lyf_nick@stu.xjtu.edu.cn (Y.L.); pengmin2020@stu.xjtu.edu.cn (M.P.)

**Keywords:** thyroid cancer, chronic illness, parenting stress, psychological distress, well-being, social support, moderated mediation

## Abstract

Women of reproductive age diagnosed with thyroid cancer face a distinctive psychological burden arising from the concurrent demands of disease management and primary caregiving, yet the mechanisms linking parenting stress to diminished well-being in this group remain poorly characterized. The present study applied a stress-buffering framework to examine whether psychological distress (anxiety and depressive symptoms) statistically mediated the association between parenting stress and well-being, and whether social support attenuates these pathways. We recruited a convenience sample of 400 consecutive eligible mothers between July and December 2025 at a tertiary cancer center in China. All were of reproductive age (18–49 years), had cytologically confirmed papillary thyroid carcinoma and at least one child aged ≤12 years, and completed the survey during their initial hospitalization, before surgery. Validated instruments assessed parenting stress, anxiety and depression, social support, and well-being; analyses included bivariate correlations, mediation, and moderated mediation models. Well-being correlated inversely with parenting stress (r = −0.447, *p* < 0.01) and anxiety and depression (r = −0.573, *p* < 0.01), and positively with social support (r = 0.515, *p* < 0.01). Psychological distress partially mediated the parenting stress–well-being association, with the indirect effect constituting 44.32% of the total effect (−0.210, 95% CI: −0.321 to −0.125). Parenting stress was positively associated with HADS scores (β = 0.322, *p* < 0.001), though this association was weaker at higher levels of social support (interaction β = −0.106, *p* = 0.006). Both parenting stress (β = −0.148, *p* = 0.001) and HADS scores (β = −0.367, *p* < 0.001) were independently associated with lower well-being, while social support moderated the parenting stress–well-being association (interaction β = 0.098, *p* = 0.003). In conclusion, parenting stress and psychological distress were jointly associated with diminished well-being in this population, but social support emerged as a modifiable protective correlate. Psychosocial interventions targeting stress reduction, emotional regulation, and support mobilization may thus hold particular promise for improving outcomes among young women with thyroid cancer.

## 1. Introduction

Living with a chronic physical illness goes beyond purely bodily symptoms. Patients facing a long-term condition need to keep making emotional and psychological adjustments as they grapple with uncertainty, shifts in social roles, and the burden of daily health management. Because of its high survival rate and low mortality, thyroid cancer is frequently classified as the malignancy whose clinical course most closely approximates a chronic disease. Nevertheless, patients need lifelong monitoring and thyroid hormone therapy, while also enduring the persistent psychological stress tied to a cancer diagnosis-all of which demand continuous emotional adaptation (Zhang et al., 2025; Wang et al., 2025; Gao et al., 2025). Globally, thyroid cancer ranks as the most common endocrine malignancy, with China alone contributing more than half of new cases (Zhang et al., 2025). Women of childbearing age face particularly high risk, likely reflecting estrogen exposure, reproductive history, and emotional stress during this life stage (Wang et al., 2025; Gao et al., 2025). Apart from biological susceptibility, these women often encounter distinct psychosocial challenges. Under traditional cultural expectations, Chinese women in this demographic usually assume primary childcare responsibilities while balancing family, financial, and occupational demands, which is accompanied by elevated parenting stress, anxiety, and depression (Sonfield et al., 2013; Zhao et al., 2024).

In contrast to many other malignancies, thyroid cancer disproportionately affects women of reproductive age (30–45 years). The standard treatments-surgery and radioactive iodine-create unique risks to fertility, pregnancy timing, and breastfeeding capacity. Because these sequelae overlap with peak child-rearing demands, mothers with thyroid cancer face a parenting stress pattern rarely seen in breast or cervical cancer groups. Whether a patient weathers this period successfully depends less on the stressors themselves than on the emotional support and social resources available to her, suggesting that social support may play an important role (Lakey & Orehek, 2011; Li et al., 2025; Sanchez-Sanchez et al., 2025).

Drawing on Cohen and Wills (1985), social support is thought to relate directly to health outcomes and to serve as a moderating resource that weakens the association between stressors and psychological well-being. For young women with thyroid cancer, parenting stress represents a major psychosocial burden stemming from the dual load of cancer treatment and child-rearing demands. We therefore hypothesized that higher social support would attenuate the associations of parenting stress with anxiety/depression and general well-being, consistent with the buffering model (Cohen & Wills, 1985; Ma & Guo, 2025).

Studies indicated that women of reproductive age report lower levels of well-being relative to men, a disparity connected to biological roles such as pregnancy, childbirth, and breastfeeding, as well as increasing social and occupational demands (He et al., 2023; Sonfield et al., 2013). These combined strains can erode psychological well-being, with downstream consequences for physical health reported in previous studies (Lin et al., 2025). Mediation analysis provides a valuable approach to uncovering these underlying mechanisms, while moderated mediation further allows exploration of protective factors, such as social support, that may buffer adverse effects. Within this framework, anxiety and depression were specified as mediators rather than confounders: stress-and-coping theory treats emotional distress as the psychological response elicited by a stressor, transmitting part of the stressor’s association with well-being, whereas a confounding account would require distress to precede and produce parenting stress; this is an ordering inconsistent with the theory guiding this study (Cohen & Wills, 1985). The hypothesized mediating mechanism follows directly from this framework. For mothers undergoing treatment for thyroid cancer, parenting stress constitutes a chronic and largely inescapable stressor: caregiving demands cannot be set aside during illness, and the resulting sustained threat appraisal is expected to give rise to anxiety and depressive symptoms (Lazarus & Folkman, 1984). Once present, such emotional distress is likely to erode general well-being by consuming the cognitive and emotional resources on which positive functioning and life evaluation depend. Social support, including the emotional support provided by family, peers, and health professionals, is theorized to enter this chain by weakening the translation of parenting stress into emotional distress, which is precisely the pathway specified in the present moderated mediation model. Two facts are already well established in the current literature: higher parenting stress and psychological distress are consistently associated with diminished happiness and life satisfaction (Bagade et al., 2023; Salafia et al., 2025), and social support can buffer the association between stress and health outcomes (Cohen & Wills, 1985). What remains unknown is whether and how these mechanisms operate in women of childbearing age with thyroid cancer, which is a population facing the dual burden of cancer treatment and child-rearing. Specifically, it remains unclear how parenting stress relates to well-being in this group, whether anxiety and depression mediate this association, and whether social support moderates these pathways (Rogusz & Harding, 2025). The present study addresses all three gaps.

Although the cross-sectional design precludes definitive causal inference, this study was designed as an exploratory mechanism investigation to generate hypotheses for future longitudinal and intervention research. As Preacher and Hayes (2008) note, cross-sectional mediation analysis serves as a critical first step in identifying potential psychosocial pathways that need to be tested in future studies. The primary aim was not to establish causality, but to clarify whether social support works as a modifiable buffer in the parenting stress, anxiety/depression, well-being nexus, thereby informing targeted nursing interventions.

This study aimed to investigate the associations among parenting stress, anxiety and depression, general well-being, and social support in women of childbearing age with thyroid cancer, and to test a moderated mediation model.

## 2. Materials and Methods

### 2.1. Study Design and Participants

The study was conducted between July and December 2025 at Sichuan Cancer Center & Institute, China. We included women of reproductive age (18–49 years) with papillary thyroid carcinoma confirmed by preoperative fine-needle aspiration (FNA) cytology and classified as category 4a–4c on preoperative ultrasound according to the Chinese Thyroid Imaging Reporting and Data System (C-TIRADS). Eligible patients were scheduled to undergo thyroid lobectomy with central lymph node dissection, had at least one child aged ≤12 years, could communicate and provide informed consent, and had no history of mental illness. For each participant, we also recorded the number and ages of her children and the household composition. We assessed all participants during their initial hospitalization, before their first surgical treatment. The interval between cytological confirmation of the diagnosis and assessment ranged from 16 to 53 days. None of the participants had other organic comorbidities or psychiatric symptoms, none were receiving psychotropic medication, none were pregnant at the time of assessment, and all were aware of their confirmed diagnosis. We excluded those with a diagnosis of other malignancies, significant communication barriers, or refusal to participate. In 2023, the Sichuan Cancer Hospital Ethics Committee approved the protocol (SCCHEC-02-2023-004), and we conducted the study following the Declaration of Helsinki and applicable national guidelines. We secured informed consent from every participant prior to data collection.

### 2.2. Measurements

The survey comprised five sections, covering sociodemographic characteristics, parenting stress, general well-being, anxiety and depression, and social support. All instruments employed were validated Chinese versions and were filled out by participants on their own concurrently.

Sociodemographic and Parenting-Related Information: We gathered sociodemographic information through questionnaire items covering age, education level, occupation, annual household income, and marital status. Parenting-related items covered the number of children, the age of each child, and household composition (nuclear family; extended three-generation household; or not living with the child).

To gauge parenting stress (independent variable), we administered the Chinese adaptation of the Parenting Stress Index (PSI). Abidin (1990) first devised the instrument, and Ren (1995) later adapted it for use in Chinese populations (Abidin, 1990; Ren, 1995). This 36-item measure uses a 5-point Likert response format (1 = strongly disagree, 5 = strongly agree). Totals can fall anywhere between 36 and 180, with higher scores pointing to heavier maternal stress. The Chinese adaptation was validated in parents of children aged 1 month to 12 years (Ren, 1995); all children of the present participants were within this age range, so the instrument was applicable to the entire sample. We adopted the conventional thresholds: normal (≤85), critical (86–90), high (91–98), and very high (≥99). In Chinese samples, the PSI has consistently reached Cronbach’s α above 0.80.

General Well-Being (Dependent Variable): In light of the documented psychological strain and quality-of-life deficits in thyroid cancer patients, the General Well-Being Scale (GWBS) was chosen to assess general well-being in this clinical population (Duan, 1996). The GWBS comprises 33 items spanning six domains (health concerns, energy, life satisfaction, mood, self-control, relaxation/tension), each scored on a Likert scale. Total scores range from 18 to 120; with higher scores indicating better well-being. Well-being is categorized as very low (≤60), low (61–72), medium (73–84), high (85–96), or very high (≥97). Previous research has found the GWBS to have satisfactory reliability and validity in Chinese.

HADS Score (Mediators): We measured anxiety and depression (mediators) with the Chinese version of the Hospital Anxiety and Depression Scale (HADS) (Zigmond & Snaith, 1983; Sun et al., 2017). This instrument contains 14 items (the anxiety and depression subscales each contain 7 items), each using a 4-point Likert response format (0–3). Either subscale yields scores between 0 and 21. We applied the standard thresholds: normal (0–7), borderline (8–10), or clinically significant (≥11). We treated scores of 8 or above on either subscale as evidence of symptomatic distress. Cronbach’s α for the Chinese adaptation generally exceeds 0.80. Because the HADS was designed as a screening instrument for emotional distress in general medical populations, its total score was used in the main analysis as a global index of psychological distress, and the HADS-A and HADS-D subscales were examined as separate mediators only in the sensitivity analysis (Section 3.4). Accordingly, the mediator is hereafter referred to as psychological distress (anxiety/depression).

Social Support (Moderator): We captured social support (moderator) using Xiao’s Social Support Rating Scale (SSRS) (Xiao, 1994). Its 10 items span three facets: objective support (3 items), subjective support (4 items), and utilization of support (3 items). Scores run from 12 to 66, with larger totals indicating stronger perceived social support. We classified these as low (≤22), medium (23–44), or high (≥45). The Chinese adaptation has performed well psychometrically.

All assessment scores and sociodemographic information were gathered at one time through a single survey administration, thereby ensuring aligned and thorough data collection for analysis.

### 2.3. Sample Size and Statistical Power

Because the primary analyses involved a moderated mediation model, the adequacy of the sample size was evaluated against the most complex regression equation rather than by general rules of thumb. This equation (the second stage of the moderated mediation analysis) contained ten predictors: six covariates (age, education level, occupation, annual household income, marital status, and number of children), parenting stress, anxiety/depression, social support, and the parenting stress × social support interaction term. An a priori power analysis was conducted with G*Power 3.1 (F tests; linear multiple regression: fixed model, R^2^ increase), the procedure that evaluates the incremental variance uniquely explained by the tested predictor over and above the remaining predictors. The analysis targeted the parenting stress × social support interaction term, with one tested predictor and ten predictors in total. Detecting a conservatively small incremental effect (f^2^ = 0.02; Aguinis et al., 2005) with α = 0.05 and power (1 − β) = 0.80 requires at least 395 valid participants. Of the 418 questionnaires collected, 18 were excluded during data quality control, leaving 400 valid cases (effective response rate 95.69%), which exceeds the required minimum.

### 2.4. Data Collection and Quality Control

We recruited a convenience sample of consecutive eligible inpatients before surgery. Trained investigators guided participants as they completed questionnaires independently, either on paper or via an online QR-code form. To safeguard data quality, we excluded questionnaires with missing values, obvious logical inconsistencies (e.g., implausible age), or completion times below one-third of the average. The final response rate was 95.69%.

### 2.5. Statistical Analysis

**Figure 1:**
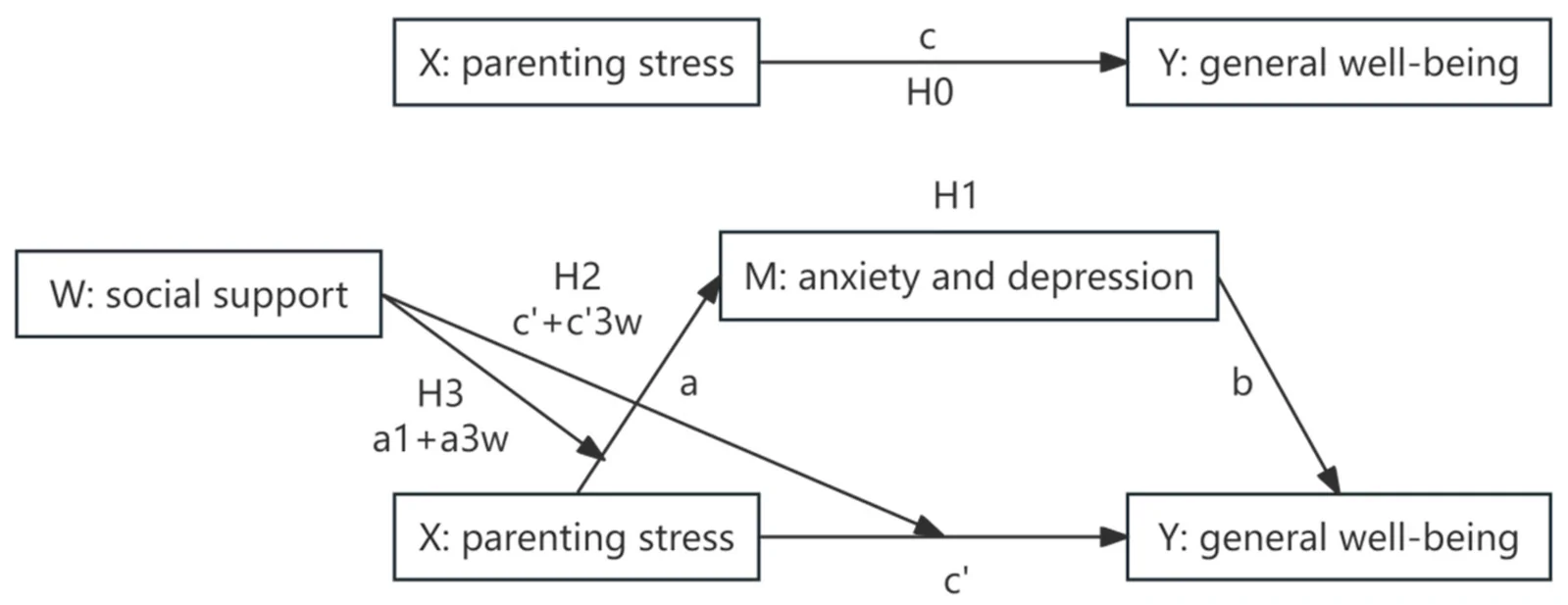
Conceptual model of the mediating effect. Notes: 1. c: parenting stress → general well-being. 2. a: parenting stress → anxiety and depression. 3. b: anxiety and depression → general well-being. 4. c′: parenting stress → general well-being. 5. c′ + c′3w: the moderating effect of social support on parenting stress and general well-being. 6. a1 + a3w: the moderating effect of social support on parenting stress and anxiety and depression.

All statistical analyses were carried out in SPSS version 26.0 (IBM Corp., Armonk, NY, USA). We summarized categorical variables by frequencies and percentages; continuous variables appeared as means and standard deviations. As a preliminary check for common method bias, we ran Harman’s single-factor test and treated a first factor variance under 40% as acceptable (Zhou & Long, 2004). We also used Pearson correlation to examine bivariate associations among parenting stress, general well-being, HADS score, and social support. For the mediation and moderated mediation analyses, we relied on Hayes’ PROCESS macro (version 4.1). Model 4 was used to test for mediation effects, and Model 8 for moderated mediation. As a sensitivity analysis, we additionally fitted a parallel mediation model (PROCESS Model 4) in which the anxiety (HADS-A) and depression (HADS-D) subscales entered simultaneously as two mediators, using the same covariates and bootstrap procedure. To reduce multicollinearity and to yield coefficients in a common metric, all continuous variables were standardized prior to model estimation, and the interaction terms were computed from these standardized variables. Accordingly, all regression coefficients reported in the text, tables, figures, and Supplementary Materials are standardized coefficients (β), and bootstrap confidence intervals and conditional effects are expressed in the same standardized metric. All regression models adjusted for six sociodemographic covariates chosen a priori on theoretical and clinical grounds: age, education, occupation, annual family income, marital status, and number of children. We evaluated the significance of indirect and moderated mediation effects through 5000 bootstrap resamples, producing bias-corrected 95% confidence intervals. Prior to the main analyses, the distributional properties of the main variables were examined. Skewness ranged from −0.456 to 0.732 and kurtosis from 0.105 to 1.615, all within the commonly accepted limits (absolute skewness < 2, absolute kurtosis < 7) (West et al., 1995), and all pairwise correlations among the main variables were below 0.60 in absolute value, supporting the use of parametric tests. Regression diagnostics were examined for both equations of the moderated mediation model. Variance inflation factors ranged from 1.01 to 1.59 (tolerances 0.63–0.99), indicating no meaningful multicollinearity. Standardized residuals exceeded ±3 in five cases (mediator equation) and two cases (outcome equation). Homoscedasticity was assessed by inspecting plots of standardized residuals against fitted values and formally with Breusch–Pagan tests; residual variance was not fully constant in either equation (mediator equation: χ^2^(9) = 28.28, *p* = 0.001; outcome equation: χ^2^(10) = 34.58, *p* < 0.001). Because all inferential tests relied on 5000 bias-corrected bootstrap resamples generated by case resampling, which do not assume residual normality or homoscedasticity, these violations do not alter the conclusions; the index of moderated mediation excluded zero (0.039, 95% CI: 0.002 to 0.079). No case exceeded a Cook’s distance of 1. A two-tailed *p*-value below 0.05 indicated statistical significance. Figure 1 illustrates the conceptual model.

## 3. Results

### 3.1. Participant Characteristics

**Figure 2:**
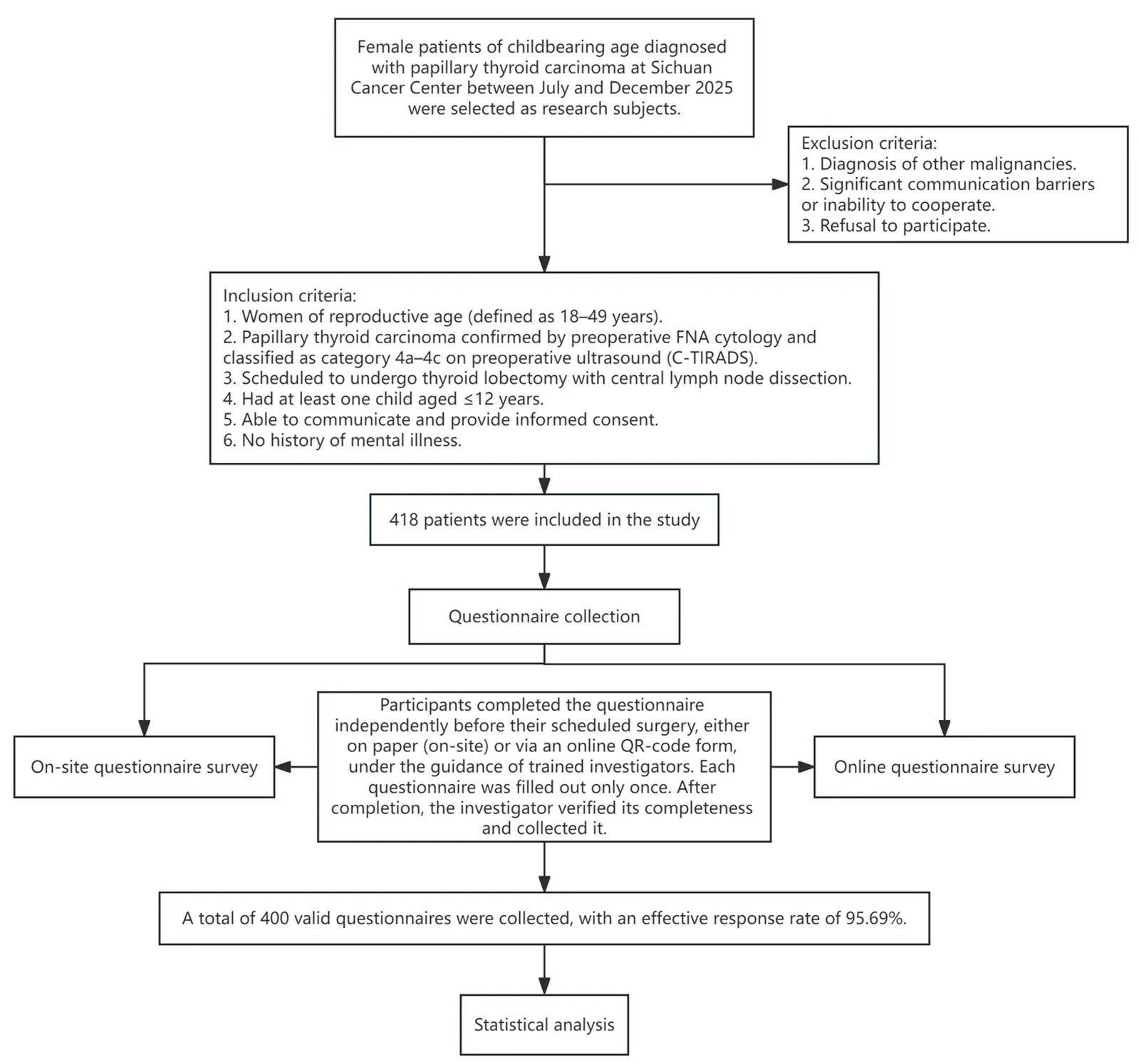
Participant recruitment flowchart.

A total of 418 women of childbearing age diagnosed with papillary thyroid cancer were recruited for this study (Figure 2). The questionnaires with the following problems were eliminated: (1) missing values; (2) obvious logical contradictions (such as filling in “150 years old” for age); (3) too short a response time (less than 1/3 of the average response time). Finally, 400 valid questionnaires were retained, yielding an effective response rate of 95.69%. Among the participants, 79.5% were employed and 94.0% were married. All were mothers with at least one child: 60.0% had one child, 37.5% had two, and 2.5% had three or more. The youngest child was aged ≤3 years in 45.0% of the families, 4–6 years in 32.5%, and 7–12 years in 22.5%, and no child was older than 12 years. Most participants lived with their children in a nuclear (59.0%) or an extended three-generation household (37.0%), while 4.0% did not live with their children. Preoperative ultrasound risk was categorized using C-TIRADS; participants were classified as category 4a, 4b, or 4c. The majority had completed at least junior high school, with 52.1% holding a college degree or higher, and 57.0% reporting an annual family income exceeding 60,000 RMB. Parenting stress was 88.18 ± 23.21, HADS score 12.81 ± 6.78, general well-being 69.07 ± 13.70, and social support 38.23 ± 8.84. Further analysis indicated that parenting stress differed significantly by annual family income (F = 7.133, *p* = 0.001) and education level (F = 3.968, *p* = 0.002), with higher levels of stress reported among participants with lower income and lower educational attainment (Table 1). There were no significant differences in parenting stress by occupation, marital status, or number of children.

### 3.2. Assessment of Common Method Bias and Instrument Reliability

Harman’s single-factor test revealed that the first factor explained 26.53% of the total variance, below the 40% cutoff. Given the limited sensitivity of this test, which can only detect severe method effects, we interpret this result as showing no pronounced single-factor structure in the data rather than as evidence that common method bias was absent. All key instruments proved highly reliable in this sample. Cronbach’s alpha reached 0.849 on the GWBS and 0.802 on the SSRS. The HADS showed 0.871 overall (0.834 for anxiety, 0.736 for depression), while the PSI yielded 0.956 overall (0.885 for parental distress, 0.920 for dysfunctional parent–child interaction, and 0.912 for child-related difficulties).

### 3.3. Correlation Analysis

Parenting stress was positively correlated with HADS score (r = 0.449, *p* < 0.01), and negatively correlated with both general well-being (r = −0.447, *p* < 0.01) and social support (r = −0.434, *p* < 0.01). HADS scores were negatively correlated with general well-being (r = −0.573, *p* < 0.01) and social support (r = −0.397, *p* < 0.01). General well-being was positively correlated with social support (r = 0.515, *p* < 0.01) (Table 2).

### 3.4. Mediating Effect Analysis

**Figure 3:**
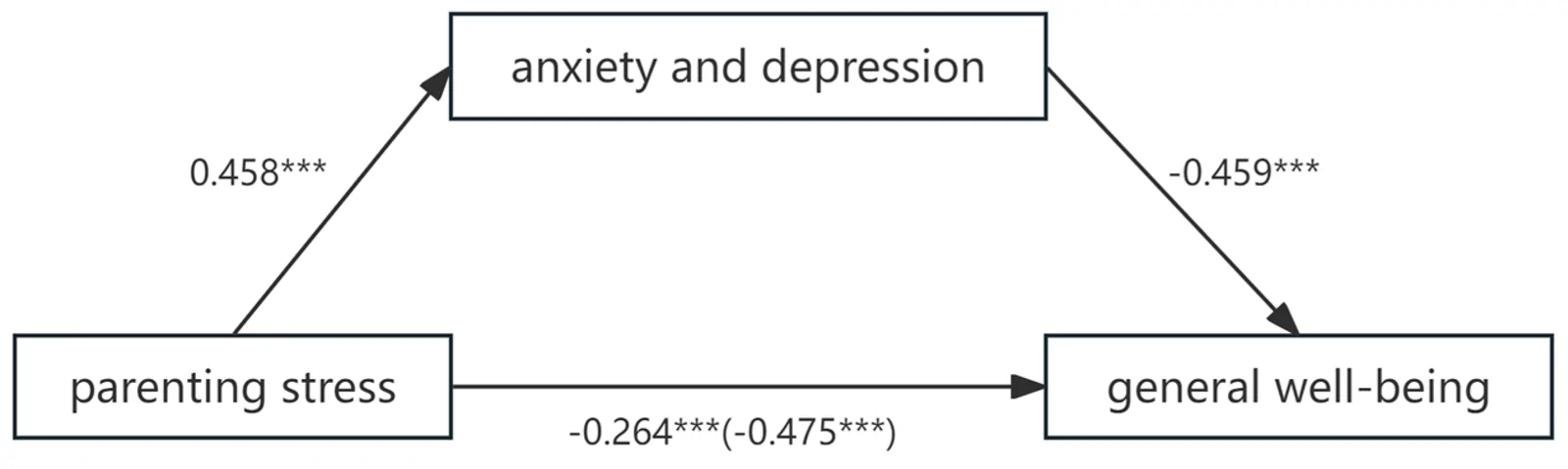
*** *p* < 0.001. Mediating effect diagram of parenting stress on general well-being. Values shown on the paths are standardized regression coefficients (β). Anxiety and depression were modeled as a single global psychological distress score (HADS total).

Mediation analysis demonstrated that HADS scores partially mediated the association between parenting stress and general well-being (Table 3, Figure 3). In Model 1, parenting stress showed a significant negative association with general well-being (β = −0.475, t = −10.385, *p* < 0.001). In Model 2, parenting stress was positively associated with HADS score (β = 0.458, t = 9.892, *p* < 0.001). In Model 3, both parenting stress (β = −0.264, t = −5.837, *p* < 0.001) and HADS score (β = −0.459, t = −10.395, *p* < 0.001) were significantly and negatively associated with general well-being, indicating that HADS scores statistically mediated the association. Bootstrap analysis further supported the mediation: the indirect effect was −0.210 (BootSE = 0.050, 95% CI: −0.321, −0.125), and the direct effect was −0.264 (BootSE = 0.045, 95% CI: −0.353, −0.175); neither confidence interval included zero. The mediation accounted for 44.32% of the total association (Table 4).

Sensitivity analysis. When the HADS-A and HADS-D subscales were entered as two parallel mediators (Supplementary Tables S1 and S2), parenting stress was positively associated with both anxiety (β = 0.443, t = 9.494, *p* < 0.001) and depression (β = 0.399, t = 8.356, *p* < 0.001), and each was negatively associated with general well-being (anxiety: β = −0.363, t = −6.443, *p* < 0.001; depression: β = −0.137, t = −2.475, *p* = 0.014). Both specific indirect effects were significant: −0.161 (BootSE = 0.044, 95% CI: −0.255, −0.084) through anxiety and −0.054 (BootSE = 0.028, 95% CI: −0.113, −0.004) through depression, accounting for 33.92% and 11.46% of the total effect, respectively. The direct effect of parenting stress remained significant (β = −0.259, t = −5.740, *p* < 0.001), and the total indirect effect (−0.215, 95% CI: −0.328, −0.126) was nearly identical to that estimated with the combined HADS score, corroborating the robustness of the mediation. Finally, the assumption of no exposure–mediator interaction was verified by adding the parenting stress × anxiety/depression interaction term to the outcome equation; the term was not significant (β = −0.030, t = −0.858, *p* > 0.05; Supplementary Table S3), supporting the specification of the mediation model. To examine whether the observed mediation was attributable to conceptual overlap between the Parental Distress subscale and the symptom measure, the mediation model was re-estimated separately for the two child-specific subscales of the PSI, retaining the same mediator and covariates. The indirect effect through emotional distress was significant for both the Parent-Child Dysfunctional Interaction subscale (effect = −0.190, 95% bootstrap CI [−0.278, −0.113]) and the Difficult Child subscale (effect = −0.200, 95% bootstrap CI [−0.309, −0.118]), a pattern consistent with that obtained for the PSI total score. These results indicate that the mediating role of emotional distress is driven by child-specific stressors rather than by shared construct content between the parental distress subscale and the symptom measure (Supplementary Table S4).

### 3.5. Moderated Mediation Effect Analysis

Further analysis using the PROCESS macro (adjusting for age, education level, occupation, annual family income, marital status, and number of children) tested whether social support moderated the pathways shown in Table 5. In the HADS-score equation, higher parenting stress was associated with higher HADS scores (β = 0.322, t = 6.391, *p* < 0.001), whereas higher social support was associated with lower scores (β = −0.180, t = −3.342, *p* < 0.001). The parenting stress × social support interaction was also significant (β = −0.106, t = −2.757, *p* = 0.006), indicating that the association between parenting stress and HADS score varied with the level of social support: the positive association was progressively weaker at higher support levels, a pattern consistent with a buffering role of social support in the association between parenting stress and anxiety/depression (R^2^ = 0.273, F = 16.306, *p* < 0.001).

In the well-being equation, parenting stress (β = −0.148, t = −3.289, *p* = 0.001) and HADS score (β = −0.367, t = −8.527, *p* < 0.001) were negatively associated with general well-being, whereas social support was positively associated with it (β = 0.238, t = 5.116, *p* < 0.001). The parenting stress × social support interaction was likewise significant (β = 0.098, t = 2.981, *p* = 0.003), indicating that social support also moderated the parenting stress–well-being association, with the negative association attenuated at higher support levels (R^2^ = 0.476, F = 35.263, *p* < 0.001). The two interaction terms accounted for an additional 1.4% and 1.2% of the variance in HADS scores and general well-being, respectively (Table 5).

Finally, to verify that social support did not also moderate the second stage of the indirect pathway, the full moderated mediation model (PROCESS Model 59) was estimated, with social support allowed to moderate the a path, the b path, and the direct path simultaneously. The anxiety and depression × social support interaction on general well-being was not significant (β = 0.003, SE = 0.039, t = 0.078, 95% CI [−0.073, 0.079], *p* > 0.05), whereas the parenting stress × social support interactions on the a path (β = −0.106, *p* < 0.01) and on the direct path (β = 0.097, *p* < 0.01) remained significant. Social support thus buffers the upstream pathways from parenting stress to emotional distress and to well-being, but does not alter the strength of the association between emotional distress and well-being, supporting the Model 8 specification used in the primary analysis (Supplementary Table S5).

**Figure 4:**
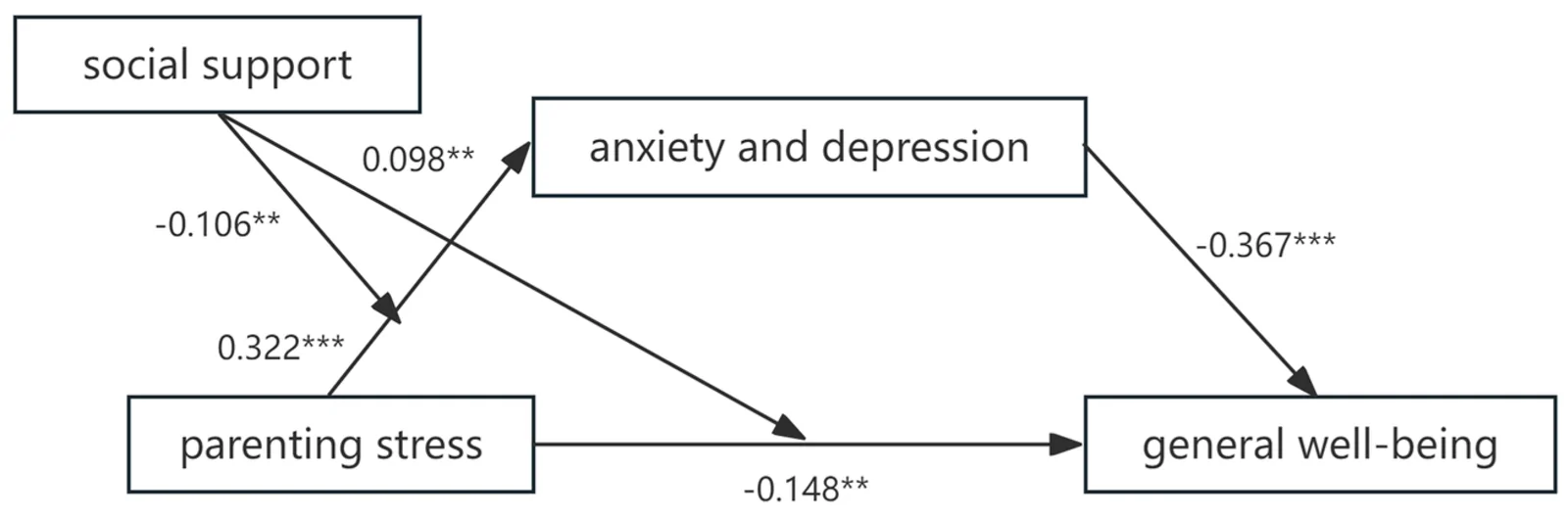
** *p* < 0.01, *** *p* < 0.001. The final moderated mediation model. The link associating the parenting stress and general well-being with anxiety and depression is moderated by social support among women with thyroid cancer at childbearing age. All path values are standardized regression coefficients (β). Anxiety and depression were modeled as a single global psychological distress score (HADS total).

Further analysis examined the mediating effect of HADS score at different levels of social support (Table 6). When social support was low (M − 1SD), the indirect effect of parenting stress on general well-being via HADS score was −0.157 (BootSE = 0.038, 95% CI: −0.245, −0.096); at the mean level of social support, the effect was −0.118 (BootSE = 0.040, 95% CI: −0.212, −0.056); and when social support was high (M + 1SD), the effect was −0.079 (BootSE = 0.050, 95% CI: −0.196, 0.003). The index of moderated mediation was 0.039 (BootSE = 0.019, 95% CI: 0.002, 0.079), supporting the moderated mediation pattern (Figure 4). In other words, when social support was high, the indirect pathway from parenting stress to well-being through anxiety/depression was no longer detectable.

### 3.6. Slope Analysis

**Figure 5:**
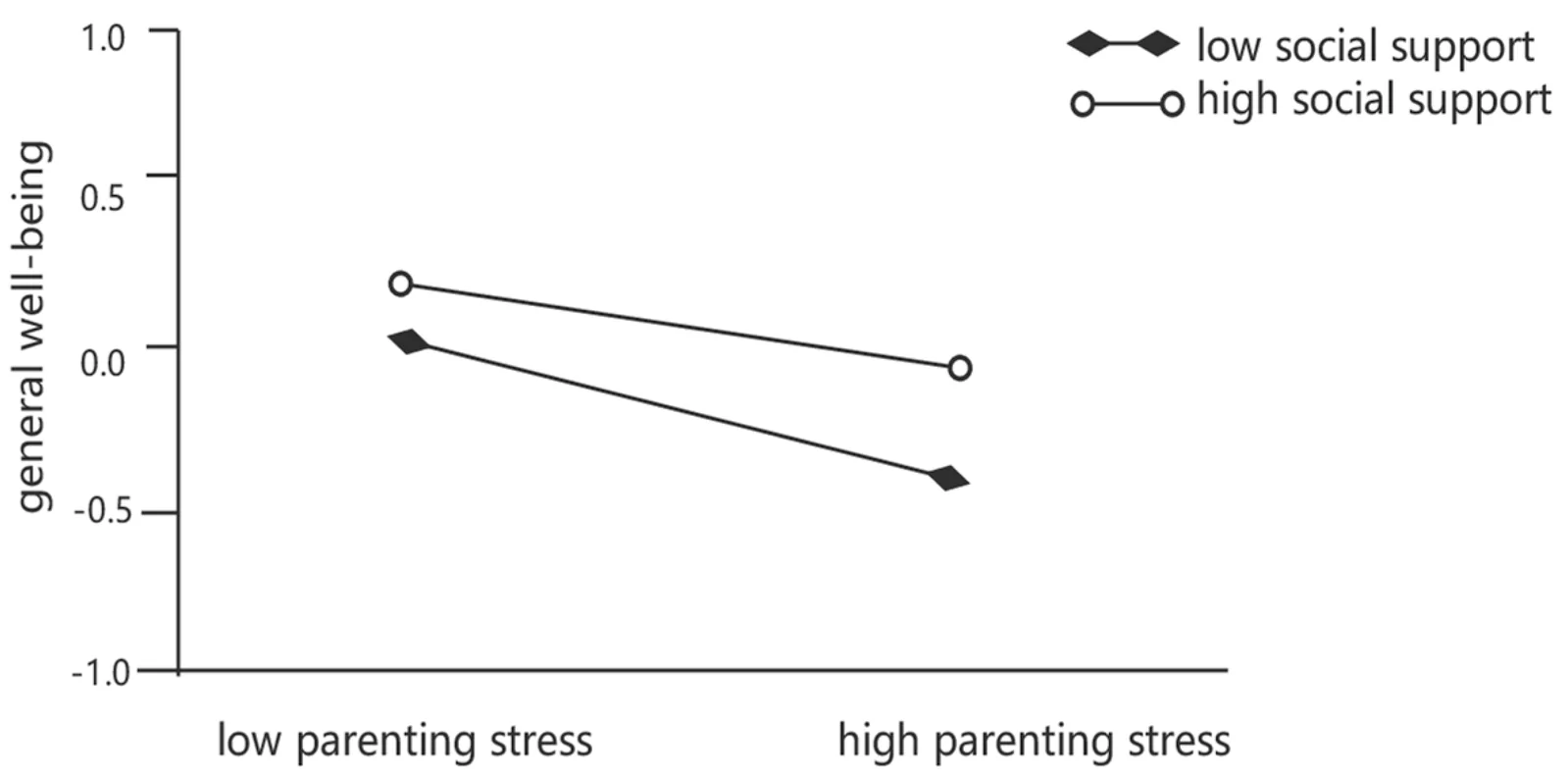
The moderating role of social support in the parenting stress–general well-being link.

**Figure 6:**
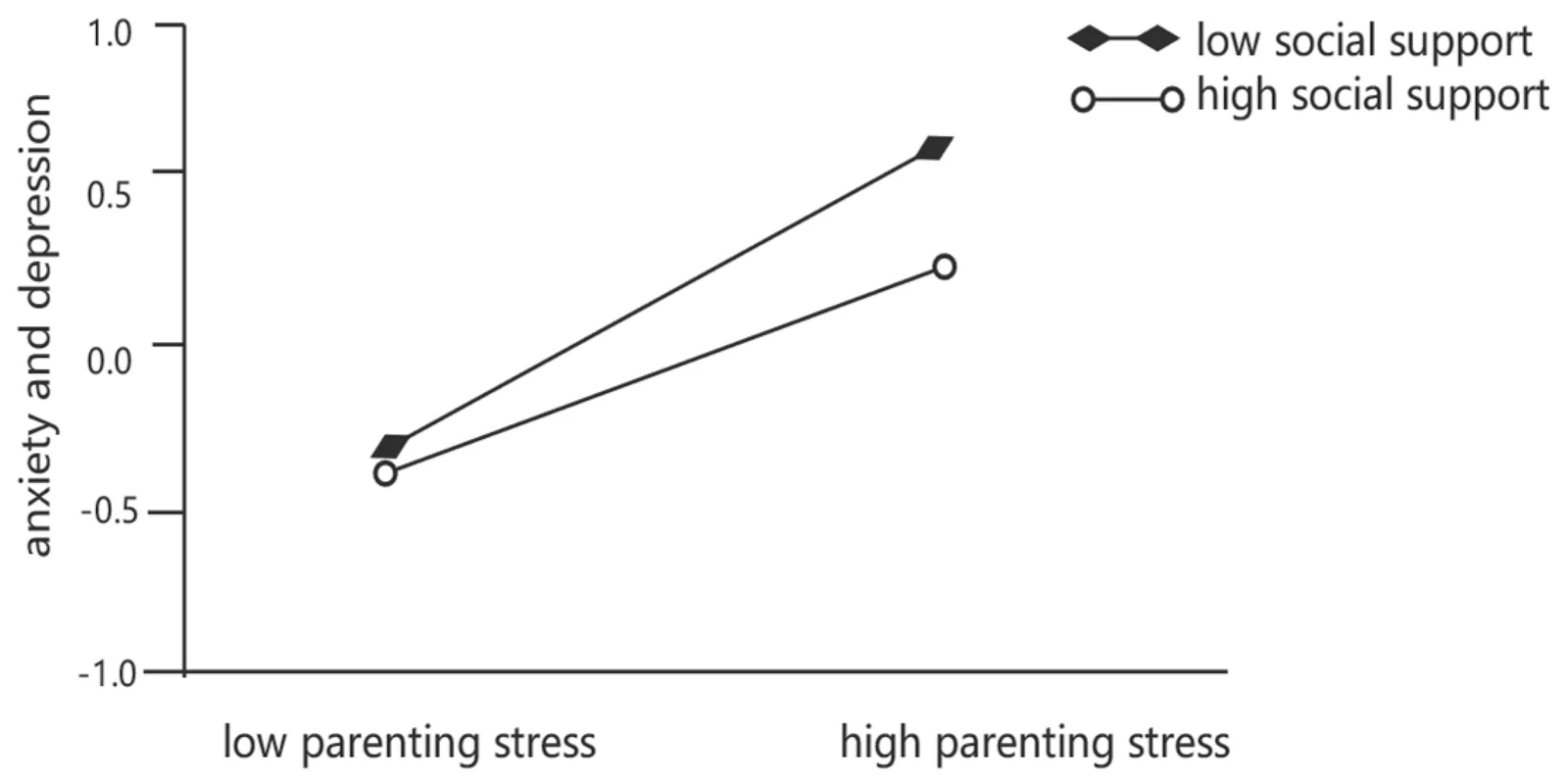
Social support as a moderator of the parenting stress–anxiety and depression association.

Simple slope analysis further illustrated the moderating effect of social support (Figure 5 and Figure 6). When social support was low, the negative association between parenting stress and general well-being was strong (simple slope = −0.247, t = −4.730, *p* < 0.001); when social support was high, this association was not significant (simple slope = −0.050, t = −0.839, *p* = 0.402). Similarly, the positive association between parenting stress and HADS score was stronger at low levels of social support (simple slope = 0.428, t = 7.458, *p* < 0.001) and weaker but still significant at high levels (simple slope = 0.216, t = 3.139, *p* = 0.002).

## 4. Discussion

This study found elevated parenting stress among reproductive-age women recently diagnosed with thyroid cancer and awaiting surgery, with lower general well-being associated with this stress both directly and indirectly through higher HADS scores. Higher parenting stress was observed among participants with lower educational attainment and lower household income. At the same time, the associations of parenting stress with psychological distress and general well-being were weaker at higher levels of social support. The results underscore why clinical care for this vulnerable group should integrate emotional health management with social support strengthening.

The observed strong links among parenting stress, HADS score, and well-being parallel the systemic, multidimensional model of parenting stress outlined in the recent literature (Rusu et al., 2025). This model highlights the interplay of family function, social support, and parenting efficacy, all converging to shape the unique psychosocial landscape of mothers facing cancer. Our results build upon this model by empirically showing that HADS scores act as a partial mediator between parenting stress and well-being, accounting for nearly 45% of the total effect. Whether this mediating pattern reflects the neuroendocrine and immunological pathways proposed in the previous literature (Cai & Gou, 2024; L. Chen et al., 2024) cannot be determined here, as no biological indicators were measured; we cite these mechanisms only as hypotheses generated by earlier work. Higher parenting stress was therefore linked to greater psychological distress and, through it, to lower well-being. Although the cross-sectional design cannot establish the direction of these pathways, this pattern suggests that early identification and intervention may be worthwhile.

As with previous research, this study also suggests that women with thyroid cancer in this age group are particularly vulnerable to a bidirectional cycle of “disease-childcare” stressors (Cai & Gou, 2024; L. Chen et al., 2024). The dual stress of cancer survivorship and intensive parenting may be accompanied by elevated anxiety and depressive symptoms, with potential repercussions for both physical and emotional health. Our data fit this picture, showing strong links among parenting stress, HADS score, and well-being, and underscoring the importance of comprehensive psychosocial assessment in clinical practice.

Especially noteworthy, our study builds on previous findings by showing that social support emerged as an important moderating factor. The associations of parenting stress with anxiety/depression and with general well-being were weaker at higher levels of social support (Lyu et al., 2025; Collins et al., 1993; Bedaso et al., 2021). Notably, when social support was high, the association between parenting stress and well-being was no longer statistically significant, a pattern consistent with a protective role of supportive environments. These findings reflect the multidimensional regulatory models of social support, such as the Relational Regulation Theory (Lakey & Orehek, 2011), which suggest that emotional well-being is shaped not only by the presence of support but by the quality and context of social interactions.

Socioeconomic status, especially lower income and limited education, was associated with higher parenting stress. These findings echo earlier work indicating that women of childbearing age from disadvantaged backgrounds see sharper drops in health-related quality of life following a thyroid cancer diagnosis, driven in large part by worries over fertility, their children’s welfare, and job security (C. Chen et al., 2023). Our study indicated that women earning less than 60,000 Chinese yuan per year and those with no more than primary education registered the greatest parenting stress, which points to a pressing need for tailored support in these groups.

These findings can be read through three complementary mechanisms. At the psychological level, sustained threat appraisal offers a plausible account of how parenting stress may turn into anxiety and depression: a mother undergoing cancer treatment cannot set aside her caregiving role, so the stressor rarely relents. Once this distress takes hold, it can wear away at well-being—attention stays fixed on potential threats, rumination builds, and less emotional energy remains for activities that would otherwise be rewarding. Behaviorally, emotional distress may further undermine well-being through changes in daily functioning, such as withdrawal from enjoyable activities, disrupted sleep, or reduced engagement in child care; conversely, the buffering pattern observed for social support may operate partly through behavior, as supportive family members relieve concrete caregiving tasks and facilitate rest and treatment adherence. Socially, the higher stress observed among women with lower income and educational attainment, together with the predominance of nuclear and three-generation households in our sample, suggests that the resources available to absorb parenting demands are structured by socioeconomic position and household composition; in the Chinese context, where mothers are conventionally expected to serve as primary caregivers, these structural conditions may be particularly consequential (C. Chen et al., 2023). These proposed mechanisms remain interpretations rather than verified pathways, given the cross-sectional and self-report nature of the data, but they offer concrete, testable directions for longitudinal research.

The partial mediation effect seen for anxiety and depression (accounting for 44.32%) suggests that treatment efforts need to address outside pressures while also building personal coping capacities. Because the mediation was partial, additional pathways are plausible; candidate mechanisms that were not assessed here—such as sleep disturbance and intra-family conflict—warrant examination in future longitudinal studies. Psychological resilience, as reported in previous research (Liu et al., 2025; Lv et al., 2021; Cui et al., 2025), may attenuate the emotional toll of such stress. These results point to the promise of interventions that build resilience, including mindfulness-based therapies or self-compassion training (Lyu et al., 2025; Zarbo, 2024), adapted for women managing both cancer and parenting.

Moreover, the simple slope results indicated a graded pattern: the associations of parenting stress with both anxiety/depression and general well-being weakened progressively as social support increased (Figure 5 and Figure 6). This finding fits with recent theoretical developments that go beyond the traditional main effect or buffering models of social support to multidimensional frameworks integrating dynamic regulation and relational needs (Lakey & Orehek, 2011; Sanchez-Sanchez et al., 2025). In clinical settings, this suggests that regular screening for both stress and support, and providing timely, personalized psychosocial interventions, can be vital for improving patient outcomes.

Relatedly, the prominent mediating role of emotional distress raises the practical question of whether anxiety and depressive symptoms could serve as a monitoring indicator when interventions are delivered to this population. Brief instruments such as the HADS are feasible for repeated use in oncology settings. The present findings, emotional distress carrying nearly half of the association between parenting stress and well-being, lend construct support to this idea. However, as the present data are cross-sectional and non-interventional, they cannot establish anxiety and depression as validated surrogate markers of intervention effects on well-being. Symptom levels should therefore be regarded as a candidate monitoring indicator that complements, rather than replaces, the direct assessment of well-being. Longitudinal intervention studies with repeated assessments are needed to determine whether changes in emotional distress reliably forecast changes in well-being in this population.

These findings provided practical guidance for emotional support in chronic disease care. Because the stress–distress and stress–well-being associations were weaker at higher levels of social support, regular screening at the time of diagnosis can assist in identifying women experiencing both high stress and inadequate support—a group that finds it hardest to manage alone with the demands of the illness. For these patients, support-oriented interventions-either through structured peer or family involvement or referrals to psychological services-may show greater efficacy than broad reassurance. Identifying which patients require such support, and when, is a vital first step in designing targeted interventions (Lyu et al., 2025; Bedaso et al., 2021).

Finally, the direction of the observed associations cannot be established. Although the hypothesized model, in which parenting stress is linked to well-being through anxiety and depression, was grounded in stress and coping theory, reverse or reciprocal pathways were equally plausible. Poorer well-being or greater psychological distress may heighten perceived parenting stress, and women in a worse emotional state may also perceive less available social support, so that support scores partly reflect concurrent distress rather than an external resource. Longitudinal or experimental designs were needed to disentangle these possibilities, and the present findings should be read as model-consistent associations rather than confirmed causal pathways.

### Limitations

Several limitations warrant mention. First, the cross-sectional design precludes causal inference and cannot establish the temporal ordering assumed by the mediation model. Reverse or reciprocal pathways cannot be excluded, for instance, greater distress or poorer well-being intensifying perceived parenting stress or eroding perceived support. Second, the participants were recruited as a convenience sample from a single tertiary hospital in Sichuan, which may restrict the generalizability of the findings to other settings and populations. Third, all variables were assessed by self-report questionnaires at a single time point, and Harman’s single-factor test has limited sensitivity; common method variance therefore cannot be excluded and may have inflated some of the observed associations. Relatedly, although the child-specific subscales of the PSI yielded indirect effects comparable to the total score, partial conceptual overlap among self-report measures cannot be entirely excluded, and multi-method assessments would strengthen future work in this area. Fourth, women with a history of mental illness were excluded; although this criterion protected the validity of the self-report measures, it may have introduced selection bias by leaving out those at potentially highest risk of distress, so that the observed associations may, if anything, be underestimated, and the applicability of the findings to populations with psychiatric comorbidity is limited. Fifth, several important clinical and family-related variables were not available. Definitive Tumor–Node–Metastasis (TNM) staging, which relies on postoperative histopathology, had not yet been established at the preoperative assessment; previous thyroid disease was not recorded; and primary caregiver status was not directly assessed (although 96.0% of the participants lived with their children, individual differences in caregiving responsibility could not be distinguished). Other unmeasured variables, such as personality traits or pre-morbid psychological functioning, may likewise have confounded the observed associations. Sixth, all participants were assessed during their initial hospitalization before surgery, within 16–53 days of diagnosis confirmation; this uniform, treatment-naive window minimized heterogeneity related to disease course and treatment exposure, but the findings characterize an early window of high distress around diagnosis and scheduled surgery, and whether the buffering pattern of social support persists into long-term survivorship remains to be verified. Seventh, a small number of extreme but clinically valid observations were identified; although the mediating pathway was robust to their influence, the moderation estimates were sensitive to these observations and should be interpreted with appropriate caution pending replication. Finally, no longitudinal follow-up was conducted, making it difficult to assess the long-term impact of psychosocial interventions or changing family dynamics over time. Future research should consider multi-center, longitudinal, and cross-cultural comparative studies to validate and extend these findings.

## 5. Conclusions

In summary, this study elucidates the complex interplay among parenting stress, anxiety and depression, social support, and general well-being in women of childbearing age with thyroid cancer. Our findings reveal that reducing parenting stress and strengthening social support may be associated with better general well-being in this population. In addition, the moderating effect of social support underscores the need for targeted psychosocial interventions. Given the influence of cultural norms and the potential for cross-cultural variation, future studies should explore these mechanisms in diverse settings to inform culturally sensitive prevention and intervention strategies for thyroid cancer in women of childbearing age.

## Figures and Tables

**Table 1:** Baseline Characteristics of the Study Population.

Variables	n (%)	Parenting Stress	Anxiety & Depression	General Well-Being	Social Support
Age (years)					
18–24	13 (3.3)	89.85 ± 27.83	11.54 ± 7.48	70.69 ± 13.46	38.92 ± 10.16
25–34	130 (32.5)	85.95 ± 24.56	12.73 ± 6.57	70.30 ± 14.19	38.67 ± 8.90
35–44	181 (45.3)	88.55 ± 23.67	12.67 ± 7.33	69.43 ± 13.74	38.06 ± 8.77
45–49	76 (19)	90.82 ± 18.50	13.47 ± 5.60	65.86 ± 12.50	37.75 ± 8.83
*F*		0.762	0.424	1.857	0.23
*p*		0.516	0.736	0.136	0.875
Education					
Primary school or below	55 (13.8)	95.05 ± 24.47	13.73 ± 7.55	69.13 ± 13.50	37.64 ± 9.46
Junior high school	89 (22.3)	91.81 ± 20.68	11.82 ± 5.83	70.38 ± 13.64	38.37 ± 9.25
High school	48 (12.0)	93.17 ± 24.85	14.71 ± 8.14	66.69 ± 16.65	36.98 ± 10.49
College diploma	91 (22.8)	85.67 ± 23.62	12.23 ± 6.99	69.85 ± 13.76	38.92 ± 8.58
Bachelor’s degree	108 (27.0)	82.63 ± 22.51	12.93 ± 6.23	68.38 ± 12.86	38.13 ± 7.87
Master’s degree or above	9 (2.3)	75.56 ± 11.91	11.22 ± 5.17	69.00 ± 7.07	41.11 ± 4.88
*F*		3.968	1.585	0.564	0.549
*p*		0.002	0.163	0.728	0.739
Occupation					
Employed	318 (79.5)	87.51 ± 23.20	12.77 ± 6.56	68.47 ± 13.61	38.37 ± 8.76
Unemployed	82 (20.5)	90.76 ± 23.19	12.96 ± 7.61	71.43 ± 13.89	37.66 ± 9.19
*t*		−1.129	−0.233	−1.75	0.65
*p*		0.26	0.816	0.081	0.516
Income (CNY)					
<60,000	172 (43.0)	91.55 ± 24.32	13.51 ± 7.47	69.11 ± 14.50	37.54 ± 9.66
60,001–120,000	129 (32.3)	89.32 ± 20.95	12.25 ± 5.93	68.77 ± 11.36	38.68 ± 7.95
>120,000	99 (24.7)	80.84 ± 22.61	12.31 ± 6.48	69.40 ± 15.11	38.82 ± 8.44
*F*		7.133	1.637	0.061	0.91
*p*		0.001	0.196	0.941	0.403
Marital Status					
Married	376 (94.0)	88.10 ± 23.14	12.77 ± 6.49	69.06 ± 13.65	38.25 ± 8.86
Unmarried	3 (0.8)	82.00 ± 36.86	14.33 ± 8.08	68.33 ± 4.62	37.00 ± 2.65
Divorced	16 (4.0)	92.81 ± 23.22	14.19 ± 12.08	68.31 ± 17.24	37.94 ± 10.30
Widowed	5 (1.3)	82.60 ± 25.34	10.60 ± 5.73	72.80 ± 10.08	38.20 ± 6.14
*F*		0.379	0.452	0.142	0.025
*p*		0.768	0.716	0.935	0.995
Number of Children					
1	240 (60)	86.61 ± 23.73	12.42 ± 6.81	69.42 ± 13.71	38.08 ± 8.73
2	150 (37.5)	90.20 ± 21.79	13.30 ± 6.83	68.69 ± 13.88	38.59 ± 8.99
≥3	10 (2.5)	95.50 ± 29.31	14.70 ± 4.42	66.50 ± 11.41	36.40 ± 9.91
*F*		1.621	1.178	0.308	0.372
*p*		0.199	0.309	0.735	0.690
The age of each child (years)					
≤3	180 (45)	86.88 ± 22.31	13.07 ± 6.53	68.18 ± 12.94	38.53 ± 8.04
4–6	130 (32.5)	89.87 ± 23.16	12.58 ± 6.82	69.63 ± 13.39	38.75 ± 9.07
7–12	90 (22.5)	88.32 ± 25.05	12.61 ± 7.24	70.06 ± 15.55	36.86 ± 9.95
*F*		0.626	0.25	0.722	1.418
*p*		0.535	0.779	0.486	0.244
Household composition					
Nuclear family	236 (59)	86.38 ± 23.07	12.56 ± 6.31	69.79 ± 14.18	38.77 ± 8.78
Extended three-generation household	148 (37)	90.86 ± 22.80	13.43 ± 7.52	67.73 ± 12.79	37.43 ± 8.97
Not living with the child	16 (4)	89.81 ± 27.67	10.63 ± 5.77	70.88 ± 14.54	37.56 ± 8.51
*F*		1.746	1.617	1.176	1.083
*p*		0.176	0.2	0.31	0.34

**Table 2:** Correlation analysis among the main study variables.

Variables	Parenting Stress	Anxiety& Depression	General Well-Being	Social Support
Parenting stress	1			
Anxiety & depression	0.449 **	1		
General well-being	−0.447 **	−0.573 **	1	
Social support	−0.434 **	−0.397 **	0.515 **	1

Notes: ** *p* < 0.01.

**Table 3:** The mediating effect model of HADS score between parenting stress and general well-being among women with thyroid cancer at childbearing age.

Variables	Mode 1 (General Well-Being)				Model 2 (Anxiety & Depression)				Model 3 (General Well-Being)			
	β	SE	t	95% CI	β	SE	t	95% CI	β	SE	t	95% CI
Age	−0.081	0.045	−1.812	[−0.169, 0.007]	0.015	0.045	0.326	[−0.075, 0.104]	−0.074	0.040	−1.873	[−0.152, 0.004]
Education	−0.109	0.050	−2.158 *	[−0.207, −0.010]	0.094	0.051	1.846	[−0.006, 0.194]	−0.065	0.045	−1.460	[−0.153, 0.023]
Occupation	0.102	0.045	2.296 *	[0.015, 0.190]	−0.009	0.045	−0.204	[−0.098, 0.080]	0.098	0.040	2.484 *	[0.020, 0.176]
Annual family income	−0.020	0.050	−0.406	[−0.118, 0.078]	−0.038	0.051	−0.754	[−0.138, 0.061]	−0.038	0.044	−0.853	[−0.125, 0.049]
Marital status	−0.001	0.045	−0.019	[−0.089, 0.088]	0.017	0.046	0.378	[−0.072, 0.107]	0.007	0.040	0.177	[−0.071, 0.085]
Number of children	0.014	0.045	0.310	[−0.075, 0.103]	0.037	0.046	0.802	[−0.053, 0.127]	0.031	0.040	0.770	[−0.048, 0.110]
Parenting stress	−0.475	0.046	−10.385 ***	[−0.565, −0.385]	0.458	0.046	9.892 ***	[0.367, 0.549]	−0.264	0.045	−5.837 ***	[−0.353, −0.175]
Anxiety & Depression									−0.459	0.044	−10.395 ***	[−0.546, −0.372]
*R*	0.481				0.459				0.631			
*R* ^2^	0.232				0.211				0.398			
*F*	16.869 ***				14.927 ***				32.297 ***			

Notes: * *p* < 0.05, *** *p* < 0.001. β = standardized regression coefficient; SE = standard error; 95% CI = 95% confidence interval of the regression coefficient. Model 1: the total association between parenting stress and general well-being (c). Model 2: the association between parenting stress and anxiety/depression (a). Model 3: the direct association between parenting stress and general well-being (c′) and the association between anxiety/depression and general well-being (b).

**Table 4:** Mediation effect test based on bootstrap.

Models	Effect	BootSE	BootLLCI	BootULCI	Effect Size Ratio
Total effect	−0.475	0.046	−0.565	−0.385	
Direct effect	−0.264	0.045	−0.353	−0.175	55.68%
Indirect effect	−0.210	0.050	−0.321	−0.125	44.32%

Notes: All effects are standardized coefficients. BootSE = bootstrap standard error; BootLLCI/ULCI = bootstrap lower/upper limit of the 95% confidence interval.

**Table 5:** Moderated mediation effects.

Variables	Model-1 (Anxiety & Depression)				Model-2 (General Well-Being)			
	β	SE	t	95% CI	β	SE	t	95% CI
Age	0.018	0.044	0.410	[−0.068, 0.104]	−0.077	0.037	−2.069 *	[−0.150, −0.004]
Education	0.068	0.049	1.380	[−0.029, 0.165]	−0.045	0.042	−1.071	[−0.128, 0.038]
Occupation	−0.006	0.044	−0.135	[−0.091, 0.080]	0.097	0.037	2.612 **	[0.024, 0.170]
Annual family income	−0.034	0.049	−0.690	[−0.129, 0.062]	−0.040	0.041	−0.954	[−0.121, 0.042]
Marital status	0.017	0.044	0.379	[−0.070, 0.103]	0.006	0.037	0.163	[−0.067, 0.079]
Number of children	0.045	0.044	1.008	[−0.042, 0.132]	0.017	0.038	0.444	[−0.057, 0.091]
Parenting stress	0.322	0.050	6.391 ***	[0.223, 0.421]	−0.148	0.045	−3.289 **	[−0.237, −0.060]
Anxiety & Depression					−0.367	0.043	−8.527 ***	[−0.452, −0.283]
Social support	−0.180	0.054	−3.342 ***	[−0.286, −0.074]	0.238	0.046	5.116 ***	[0.146, 0.329]
Parenting stress × Social support	−0.106	0.038	−2.757 **	[−0.182, −0.030]	0.098	0.033	2.981 **	[0.034, 0.163]
*R*	0.523				0.690			
*R* ^2^	0.273				0.476			
Δ*R*^2^	0.014				0.012			
*F*	16.306 ***				35.263 ***			

Notes: * *p* < 0.05, ** *p* < 0.01, *** *p* < 0.001. β = standardized regression coefficient; SE = standard error; 95% CI = 95% confidence interval of the regression coefficient.

**Table 6:** Conditional Indirect Effects of Parenting Stress on General Well-Being via HADS score at Different Levels of Social Support.

Social Support Level	Effect	BootSE	BootLLCI	BootULCI
M − 1SD (Low)	−0.157	0.038	−0.245	−0.096
M (Mean)	−0.118	0.04	−0.212	−0.056
M + 1SD (High)	−0.079	0.05	−0.196	0.003
Index	0.039	0.019	0.002	0.079

Note: Conditional indirect effects are expressed as standardized coefficients. M = mean; SD = standard deviation; BootSE = bootstrap standard error; BootLLCI/ULCI = bootstrap lower/upper limit of the 95% confidence interval. A 95% confidence interval that includes zero indicates a non-significant conditional indirect effect.

## Data Availability

The data supporting this study are available from the corresponding author upon reasonable request. Due to privacy and ethical restrictions related to patient confidentiality and sensitive psychosocial information (including parenting stress, anxiety/depression symptoms, and socioeconomic status), the raw dataset is not publicly available.

## Supplementary Materials

The following supporting information can be downloaded at https://www.mdpi.com/article/10.3390/bs16091491/s1. Table S1: Parallel mediation analysis with the HADS anxiety and depression subscales as simultaneous mediators; Table S2: Mediation effect test based on bootstrap; Table S3: Test of the exposure–mediator interaction: regression of general well-being on parenting stress, anxiety/depression, and their interaction term; Table S4: Supplementary analyses for the child-specific subscales of the Parenting Stress Index: correlations with emotional distress and general well-being, and mediation models with emotional distress as the mediator (N = 400); Table S5: Regression results of the full moderated mediation model (PROCESS Model 59) with social support moderating the a path, b path, and direct path (N = 400).

## Abbreviations

The following abbreviations are used in this manuscript:

BootLLCI	Bootstrap Lower Limit of the 95% Confidence Interval
BootSE	Bootstrap Standard Error
BootULCI	Bootstrap Upper Limit of the 95% Confidence Interval
CI	Confidence Interval
C-TIRADS	Chinese Thyroid Imaging Reporting and Data System
FNA	Fine-Needle Aspiration
GWBS	General Well-Being Scale
HADS	Hospital Anxiety and Depression Scale
PSI	Parenting Stress Index
SD	Standard Deviation
SSRS	Social Support Rating Scale
TNM	Tumor–Node–Metastasis
