# Supplementary material for: Psychological Assessment of Parenting Stress and Emotional Support: A Moderated Mediation Model of Psychological Distress and Well-Being Among Childbearing-Age Women with Thyroid Cancer
Source: Behav Sci (Basel). 2026 Aug 26;16(9):1491. doi: 10.3390/bs16091491 (PMC13603398; doi:10.3390/bs16091491)
Supplement: Supplementary file 1 [file behavsci-16-01491-s001.zip › behavsci-4431182-supplementary.pdf]

Supplementary Table S1. Parallel mediation analysis with the HADS anxiety and depression subscales as simultaneous mediators.

**Variables**	**Model 1 (Anxiety)**				**Model 2 (Depression)**				**Model 3 (General Well-Being)**			
	β	*SE*	*t*	*95%CI*	*β*	*SE*	*t*	*95%CI*	*β*	*SE*	*t*	*95%CI*
Age	-0.010	0.046	-0.227	[-0.100，0.080]	0.040	0.047	0.844	[-0.052，0.131]	-0.080	0.040	-2.007*	[-0.157，-0.002]
Education	0.118	0.051	2.298*	[0.017，0.219]	0.053	0.053	1.003	[-0.051，0.156]	-0.058	0.045	-1.308	[-0.146，0.029]
Occupation	-0.025	0.046	-0.548	[-0.115，0.065]	0.009	0.047	0.199	[-0.082，0.101]	0.095	0.039	2.401*	[0.017，0.172]
Annual family income	-0.050	0.051	-0.972	[-0.150，0.051]	-0.020	0.052	-0.375	[-0.122，0.083]	-0.041	0.044	-0.928	[-0.128，0.046]
Marital status	0.024	0.046	0.532	[-0.066，0.115]	0.007	0.047	0.141	[-0.086，0.099]	0.009	0.04	0.225	[-0.069，0.087]
Number of children	0.038	0.046	0.818	[-0.053，0.129]	0.030	0.047	0.626	[-0.063，0.123]	0.032	0.04	0.795	[-0.047，0.110]
Parenting stress	0.443	0.047	9.494***	[0.352，0.535]	0.399	0.048	8.356***	[0.305，0.493]	-0.259	0.045	-5.740***	[-0.348，-0.170]
Anxiety									-0.363	0.056	-6.443***	[-0.474，-0.252]
Depression									-0.137	0.055	-2.475*	[-0.245，-0.028]
R			0.444				0.403				0.636	
R^2			0.197				0.162				0.404	
F			13.776***				10.847***				29.421***	

Note. **P* < 0.05, ***P* < 0.01, ****P* < 0.001. β = standardized regression coefficient; SE = standard error; 95%CI = 95% confidence interval of the regression coefficient.

Model 1: the total association between parenting stress and general well-being (c). Model 2: the association between parenting stress and anxiety/depression (a). Model 3: the direct association between parenting stress and general well-being (c′) and the association between anxiety/depression and general well-being (b).

Supplementary Table S2. Mediation effect test based on bootstrap

Models	Effect	BootSE	BootLLCI	BootULCI	Effect size ratio
Total effect	-0.475	0.046	-0.565	-0.385	100.00%
Direct effect	-0.259	0.045	-0.348	-0.170	54.60%
Indirect effect (Total)	-0.215	0.052	-0.328	-0.126	45.38%
Indirect effect 1：Parenting stress→Anxiety→General Well-Being	-0.161	0.044	-0.255	-0.084	33.92%
Indirect effect 2：Parenting stress→Depression→General Well-Being	-0.054	0.028	-0.113	-0.004	11.46%

Note. All effects are standardized coefficients. BootSE = bootstrap standard error; BootLLCI/ULCI = bootstrap lower/upper limit of the bias-corrected 95% confidence interval (5,000 resamples). BootSE = bootstrap standard error; BootLLCI/ULCI = bootstrap lower/upper limit of the 95% confidence interval.

Supplementary Table S3. Test of the exposure–mediator interaction: regression of general well-being on parenting stress, anxiety/depression, and their interaction term.

**Variables**	**General Well-Being**			
	β	*SE*	*t*	*95%CI*
Age	-0.077	0.040	-1.929	[-0.155，0.002]
Education	-0.064	0.045	-1.418	[-0.152，0.025]
Occupation	0.096	0.040	2.424*	[0.018，0.174]
Annual family income	-0.037	0.044	-0.837	[-0.124，0.050]
Marital status	0.012	0.040	0.288	[-0.068，0.091]
Number of children	0.030	0.040	0.739	[-0.049，0.109]
Parenting stress	-0.256	0.046	-5.501***	[-0.347，-0.164]
Anxiety & Depression	-0.464	0.045	-10.419***	[-0.551，-0.376]
Parenting stress * Anxiety & Depression	-0.030	0.035	-0.858	[-0.099，0.039]
R	0.632			
R^2	0.399			
F	28.771***			

Note. **P* < 0.05, ***P* < 0.01, ****P* < 0.001. β = standardized regression coefficient; SE = standard error; 95%CI = 95% confidence interval of the regression coefficient.

Supplementary Table S4 (Part A, B, C). Supplementary analyses for the child-specific subscales of the Parenting Stress Index: correlations with emotional distress and general well-being, and mediation models with emotional distress as the mediator (N = 400).

Note. Model 1 = total-effect model (subscale → GWB); Model 2 = mediator model (subscale → HADS); Model 3 = direct-effect model (subscale + HADS → GWB). All models adjusted for the same six covariates as the primary analysis. Bootstrap CIs are bias-corrected with 5,000 resamples. $P < 0.05$, $*P < 0.01$, $**P < 0.001$.

Part A. Pearson correlation matrix among the PSI subscales, emotional distress (HADS), and general well-being (GWB).

Variables	DC	P-CDI	PD	HADS	GWB
Difficult Child (DC)	1				
Parent-Child Dysfunctional Interaction (P-CDI)	0.809**	1			
Parental Distress (PD)	0.658**	0.613**	1		
Anxiety&Depression	0.386**	0.351**	0.469**	1	
General Well-Being	-0.386**	-0.313**	-0.501**	-0.573**	1

Part B. Regression and bootstrap results of the mediation model for the Parent-Child Dysfunctional Interaction (P-CDI) subscale.

Variables	Model 1 (Anxiety)				Model 2 (Depression)				Model 3 (General Well-Being)			
	β	*SE*	*t*	*95%CI*	*β*	*SE*	*t*	*95%CI*	*β*	*SE*	*t*	*95%CI*
Age	-0.084	0.048	-1.758	[-0.178，0.010]	0.016	0.048	0.331	[-0.078，0.109]	-0.076	0.041	-1.855	[-0.156，0.005]
Education	-0.110	0.054	-2.025*	[-0.216，-0.003]	0.102	0.054	1.899	[-0.004，0.209]	-0.056	0.047	-1.206	[-0.148，0.035]
Occupation	0.093	0.048	1.959	[-0.000，0.186]	-0.001	0.047	-0.020	[-0.094，0.092]	0.093	0.041	2.281*	[0.013，0.172]
Annual family income	-0.003	0.053	-0.059	[-0.108，0.101]	-0.052	0.053	-0.979	[-0.156，0.052]	-0.030	0.046	-0.665	[-0.120，0.059]
Marital status	0.002	0.048	0.044	[-0.092，0.096]	0.014	0.048	0.295	[-0.080，0.108]	0.010	0.041	0.232	[-0.071，0.090]
Number of children	0.004	0.048	0.091	[-0.091，0.099]	0.044	0.048	0.907	[-0.051，0.138]	0.027	0.041	0.658	[-0.054，0.108]
P-CDI	-0.342	0.049	-6.921***	[-0.439，-0.245]	0.363	0.049	7.372***	[0.266，0.460]	-0.152	0.045	-3.376**	[-0.241，-0.064]
Anxiety & Depression									-0.523	0.043	-12.076***	[-0.608，-0.438]
R			0.356				0.365				0.603	
R^2			0.127				0.134				0.364	
F			8.128***				8.628***				27.968***	

Models	Effect	BootSE	BootLLCI	BootULCI	Effect size ratio
Total effect	-0.342	0.049	-0.439	-0.245	
Direct effect	-0.152	0.045	-0.241	-0.064	44.49%
Indirect effect (P-CDI → HADS → GWB)	-0.190	0.043	-0.278	-0.113	55.51%

Part C. Regression and bootstrap results of the mediation model for the Difficult Child (DC) subscale.

Variables	Model 1 (Anxiety)				Model 2 (Depression)				Model 3 (General Well-Being)			
	β	*SE*	*t*	*95%CI*	*β*	*SE*	*t*	*95%CI*	*β*	*SE*	*t*	*95%CI*
Age	-0.100	0.043	-2.322*	[-0.185，-0.015]	0.033	0.045	0.746	[-0.055，0.122]	-0.086	0.039	-2.216*	[-0.162，-0.010]
Education	-0.084	0.048	-1.746	[-0.179，0.011]	0.068	0.050	1.352	[-0.031，0.166]	-0.055	0.043	-1.276	[-0.141，0.030]
Occupation	0.114	0.043	2.634**	[0.029，0.198]	-0.018	0.045	-0.411	[-0.106，0.070]	0.106	0.039	2.730**	[0.030，0.182]
Annual family income	-0.005	0.048	-0.108	[-0.100，0.089]	-0.055	0.050	-1.095	[-0.153，0.043]	-0.029	0.043	-0.658	[-0.114，0.057]
Marital status	0.007	0.043	0.163	[-0.078，0.092]	0.010	0.045	0.224	[-0.079，0.099]	0.011	0.039	0.291	[-0.065，0.088]
Number of children	0.001	0.044	0.016	[-0.085，0.087]	0.051	0.045	1.127	[-0.038，0.140]	0.022	0.039	0.571	[-0.055，0.100]
DC	-0.519	0.043	-11.986***	[-0.605，-0.434]	0.470	0.045	10.468***	[0.382，0.559]	-0.319	0.044	-7.244***	[-0.406，-0.232]
Anxiety & Depression									-0.426	0.044	-9.740***	[-0.512，-0.340]
R			0.532				0.479				0.65	
R^2			0.283				0.229				0.423	
F			22.089***				16.625***				35.813***	

Models	Effect	BootSE	BootLLCI	BootULCI	Effect size ratio
Total effect	-0.519	0.043	-0.605	-0.434	100.00%
Direct effect	-0.319	0.044	-0.406	-0.232	61.40%

Indirect effect (DC → HADS → GWB)	-0.200	0.050	-0.309	-0.118	38.60%

Supplementary Table S5. Regression results of the full moderated mediation model (PROCESS Model 59) with social support moderating the a path, b path, and direct path (N = 400).

Variables	Model-1 (Anxiety&Depression)				Model-2 (General Well-Being)			
	β	*SE*	*t*	*95%CI*	*β*	*SE*	*t*	*95%CI*
Age	0.018	0.044	0.41	[-0.068，0.104]	-0.077	0.037	-2.064*	[-0.150，-0.004]
Education	0.068	0.049	1.38	[-0.029，0.165]	-0.045	0.042	-1.066	[-0.128，0.038]
Occupation	-0.006	0.044	-0.135	[-0.091，0.080]	0.097	0.037	2.603**	[0.024，0.170]
Annual family income	-0.034	0.049	-0.69	[-0.129，0.062]	-0.04	0.042	-0.956	[-0.122，0.042]
Marital status	0.017	0.044	0.379	[-0.070，0.103]	0.006	0.037	0.168	[-0.067，0.080]
Number of children	0.045	0.044	1.008	[-0.042，0.132]	0.017	0.038	0.439	[-0.058，0.091]
Parenting stress	0.322	0.05	6.391***	[0.223，0.421]	-0.147	0.048	-3.050**	[-0.242，-0.052]
Anxiety&Depression					-0.368	0.045	-8.272***	[-0.456，-0.281]
Social support	-0.18	0.054	-3.342**	[-0.286，-0.074]	0.238	0.047	5.108***	[0.146，0.329]
Parenting stress * Social support	-0.106	0.038	-2.757**	[-0.182，-0.030]	0.097	0.037	2.636**	[0.025，0.170]
Anxiety&Depression * Social support					0.003	0.039	0.078	[-0.073，0.079]
R			0.523				0.69	
R^2			0.273				0.476	
F			16.306***				31.976***	

Note. All coefficients are standardized (β). Model 1 = mediator model (outcome: HADS); Model 2 = outcome model (outcome: GWB). Models were estimated simultaneously within PROCESS Model 59 and adjusted for the six covariates shown. *P* < 0.05, **P* < 0.01, ***P* < 0.001.
